# A combination of ultrasound-targeted microbubble destruction with transplantation of bone marrow mesenchymal stem cells promotes recovery of acute liver injury

**DOI:** 10.1186/s13287-018-1098-4

**Published:** 2018-12-29

**Authors:** Ting Sun, Feng Gao, Xin Li, Yingyu Cai, Min Bai, Fan Li, Lianfang Du

**Affiliations:** Department of Medical Ultrasound, Shanghai General Hospital, Shanghai Jiao Tong University School of Medicine, 100 Haining Road, Shanghai, 200080 China

**Keywords:** Bone marrow mesenchymal stem cells, Ultrasound, Microbubble, Stem cell transplantation, Acute liver injury

## Abstract

**Background:**

Bone marrow mesenchymal stem cells (BMSCs) can provide an additional source of therapeutic stem cells for regeneration of liver cells during acute liver injury (ALI). However, the insufficient hepatic homing by the transplanted BMSCs limits their applications. Ultrasound-targeted microbubble destruction (UTMD) has been reported to promote the homing of transplanted stem cells into the ischemic myocardium. In this study, we investigated whether UTMD promotes the hepatic homing of BMSCs in ALI rats and evaluated the therapeutic effect.

**Methods:**

BMSCs were isolated from the femurs and tibias of Sprague-Dawley (SD) rats. The isolated BMSCs were stably transfected with a lentivirus expressing enhanced green fluorescent protein (EGFP) that can be visualized and quantified in vivo after transplantation. Both tumor necrosis factor α (TNF-α) and stromal cell-derived factor 1 (SDF-1) were used to verify the appropriate ultrasound parameters. The ALI rats were divided into four groups: control, BMSCs, UTMD, and UTMD + BMSCs. The protein and mRNA expression levels of SDF-1, intercellular cell adhesion molecule (ICAM-1), vascular cell adhesion molecule 1 (VCAM-1), hepatocyte growth factor (HGF), and monocyte chemotactic protein 1 (MCP-1) in the exposed livers were analyzed at 48 h after treatment. ALI recovery was determined by serum biochemical parameters and histology.

**Results:**

The isolated rat BMSCs demonstrated a good proliferation potential that was both osteogenic and adipogenic in differentiation and expressed cluster of differentiation (CD) 29 and CD90, but not CD45 or CD11b/c. After BMSC and/or UTMD treatment, the number of GFP-labeled BMSCs in the UTMD + BMSCs group was significantly higher than that of the BMSCs group (9.8 ± 2.3 vs. 5.2 ± 1.1/per high-power field). Furthermore, the expression of GFP mRNA was performed for evaluation of the homing rate of BMSCs in injury sites as well. In addition, the expression levels of SDF-1, ICAM-1, VCAM-1, HGF, and MCP-1 were higher (*p* < 0.01) in UTMD+BMSCs group. The serum levels of biomarkers were significantly lower in the UTMD + BMSCs group, and the apoptotic rate of hepatocytes in the UTMD + BMSCs group was markedly lower than that of the BMSCs group (all *p* < 0.05). The hepatic pathology was significantly alleviated in the UTMD + BMSCs group.

**Conclusions:**

UTMD treatment efficiently induced a favorable microenvironment for cell engraftment, resulting in improvement of hepatic homing of BMSCs, which was probably mediated through upregulation of the expression of adhesion molecules and cytokines. UTMD treatment appeared to be an effective and noninvasive approach to achieve better efficacy of BMSC-based therapy for repairing a severely injured liver.

**Electronic supplementary material:**

The online version of this article (10.1186/s13287-018-1098-4) contains supplementary material, which is available to authorized users.

## Background

Severe acute liver injury (ALI) is a syndrome caused by massive injury of hepatocytes and progressive deterioration of liver function that eventually progresses to cerebral and renal functions without intervention [1]. Generally, orthotopic liver transplantation is considered to be the most effective treatment [2]. However, the known shortage of liver donors, high expenses of surgery, and risk of graft rejection restrict liver transplantation in the clinic. Therefore, nontransplant-associated therapies of ALI should be developed [3].

Numerous studies have shown that stem cells can be used as a hepatocyte regeneration source for repairing an injured liver [4, 5]. Of stem cells, bone marrow-derived mesenchymal stem cells (BMSCs) have demonstrated therapeutic potential in treating acute and chronic liver diseases [6]. Besides its pluripotent differentiation and high self-renewal capacity, BMSCs are easier to obtain, separate, and expand, without major ethical or religious concerns compared with embryonic stem cells. Therefore, BMSCs have been explored to treat myocardial infarction, kidney injury, liver injury, and many other diseases [7, 8]. However, the ability of BMSCs to home to targeted tissues has been shown to be unsatisfactory, thus compromising the therapeutic effects. Therefore, facilitation of BMSCs homing to target tissues represents a priority for utilizing BMSCs for regenerative medicine.

Previous studies have demonstrated that ultrasound-targeted microbubble destruction (UTMD), accompanied by a jetting micro-stream resulting from sonication, is an efficient method to deliver drugs, including gene vectors, to the desired organs or tissues [9–11]. Furthermore, accumulated evidence suggests that UTMD enhances the migration of BMSCs to the infarcted myocardium and injured kidney [12, 13]. An underlying requirement for the efficient homing of BMSCs is to create a microenvironment providing a satisfactory “soil” for the mobility of BMSCs. However, it is unclear whether UTMD can efficiently facilitate the homing of BMSCs to the livers of ALI rats.

Therefore, in this study, we generated a severe ALI rat model to investigate the impact of UTMD on the homing of BMSCs to the injured liver. In addition, we explored the detailed mechanism involved in the increased cell engraftment by UTMD treatment.

## Materials and methods

### Isolation and culture of rat BMSCs

BMSCs were harvested from the bone marrow of Sprague Dawley (SD) rats (3–4 weeks old) by the adherent centrifugation method. First, each rat was sacrificed by cervical dislocation and disinfected by immersion in 75% alcohol. Then, the femurs and tibias were carefully removed, the metaphysis was removed, and the bone marrow cavity was repeatedly rinsed with Dulbecco’s modified Eagle medium (DMEM)/F12 (Gibco, Grand Island, NY, USA) until the marrow cavity became white. The collected bone marrow materials were centrifuged for 5 min. The cell sediment was resuspended with complete culture medium (containing 90% DMEM/F12, 10% fetal bovine serum (Gibco, Grand Island, NY, USA), 1% 100 U/mL penicillin, and 100 mg/mL streptomycin). The cells were cultured in a 100-mm^2^ treated cell culture dish (Corning, Grand Island, NY, USA) in a cell incubator (37 °C, 5% CO_2_), and the medium was replaced every 2–3 days. When the cells reached 70–80% confluence, they were passaged.

### Basic characteristics and differentiation of BMSCs

The BMSCs after the third passage were examined under a transmission electron microscope (TEM, CM-120, Philips, Holland). Briefly, the BMSCs were cultured at 1 × 10^4^ cells/well in six-well plates. The culture medium was replaced with osteogenic and adipogenic differentiation medium to induce differentiation once the cells became 50–60% confluent. After 2–3 weeks, the cells were fixed with 4% paraformaldehyde for 10–15 min. After rinsing with phosphate-buffered saline (PBS), the cells were stained with oil red O and alizarin Bordeaux S (Sigma-Aldrich, St. Louis, MO, USA) to identify lipid droplets and calcium nodules.

BMSCs of the third passage (2 × 10^3^ cells/well) were cultivated in a 96-well plate for the cell proliferation test. The extent of cell growth was assessed for 7 consecutive days using a cell counting kit-8 (CCK-8) assay (Dojindo, Kyushu, Japan). A 10-μL aliquot of CCK-8 solution was added into each well, and then, the cells were incubated for 2 h at 37 °C. The absorbance at 450 nm of each well was measured by a multimode reader (Thermo Fisher, New York, NY, USA).

For detection of the cell cycle, the obtained cells were cultured in a six-well plate. When the cells reached 80% confluence, the harvested cells were fixed in 75% ethanol overnight at 4 °C. Propidium iodide (BD Biosciences, San Jose, CA, USA) was used to stain DNA for 30 min at room temperature. Then, the data were analyzed using flow cytometry (BD Biosciences, AccuriC6, USA).

### Immunofluorescence staining of hallmarks of BMSCs and flow cytometric detection

The BMSCs at the third passage were harvested at a concentration of 1 × 10^9^ cells and resuspended in 100 μL of PBS. The cells in the suspension were stained with 5 μL of phycoerythrin-conjugated rat anti-cluster of differentiation (CD)11b/c, CD29, CD45, and CD90 antibodies (Biolegend, San Diego, CA, USA) or the corresponding isotype control for 30 min at room temperature. The surface markers on BMSCs were analyzed by flow cytometry. The acquired data were analyzed using Flow Jo software (BD Biosciences Beckman Coulter), and the mean fluorescence intensity was calculated.

### Transduction of BMSCs

The required amount of virus was calculated based on the formula: the amount of virus = cells × 10 (multiplicity of infection). The BMSCs were seeded in six-well plates at 1 × 10^5^/well for 24 h, transduced with lentivirus expressing enhanced green fluorescent protein (EGFP; HANBIO, Wuhan, Hubei, China) in 1 mL of fresh DMEM/F12, and incubated for 4 h. Then, 1 mL of fresh medium was added, and the cells were cultured for 20 h. Next, the old medium was removed, the cells were washed with PBS, fresh complete medium was added, and the cells were cultured for another 24 h. The transduction rate was determined by fluorescence microscopy and flow cytometry.

### Induction of severe ALI in a rat model

Male SD rats at 6-8 weeks of age were provided by the Center for Experimental Animals of Shanghai General Hospital. In this experiment, all animal protocols were carried out in accordance with the guidelines of the National Institutes of Health Guide for the Care and Use of Laboratory Animals, which were approved by the regulations set by the Ethics Committee of Shanghai General Hospital as well. Eight rats were randomly divided into the model and control groups. The rats in the model group were injected intraperitoneally with 1.5 g/kg d-galactosamine (D-Gal) (Sigma, St. Louis, MO, USA) to induce severe ALI. In this study, the ALI animal model refers to severe liver damage. The injection dose was determined according to previous animal studies [14]. Changes in liver function were monitored for 3 days, and then, the animals were sacrificed for histological evaluation.

### UTMD parameters

To select the optimal UTMD parameters, 12 rats were randomly assigned into four groups 24 h after injection of D-Gal: (1) control, (2) 1.0 W/cm^2^ UTMD (3) 1.5 W/cm^2^ UTMD, and (4) 2.0 W/cm^2^ UTMD.

The microbubbles used in this study were SonoVue (Bracco, Milan, Italy), a contrast agent that has been approved by the Food and Drug Administration (USA). A therapeutic ultrasound (Topteam161 Physioson-Basic, Elektromedizin, AG, Germany) was applied in this experiment. The rats were placed horizontally on a fixed shelf after shaving the hair from their abdomen. The ultrasound probe (diameter, 2.0 cm) was placed above the whole liver of the rat, with one layer of gel. Before ultrasound radiation, 300 μL of the microbubble suspension was injected slowly via the tail vein. The other acoustic parameters were as follows: 1 MHz, 20% duty cycle, and exposure time of 10 min, which was determined based on previous literature examples [15, 16]. The rats were sacrificed at 48 h after UTMD, and the collected livers were stored in liquid nitrogen. The hepatic expression of stromal cell-derived factor 1 (SDF-1) and tumor necrosis factor α (TNF-α) was determined.

### Study design for animal experiments

**Fig. 1:**
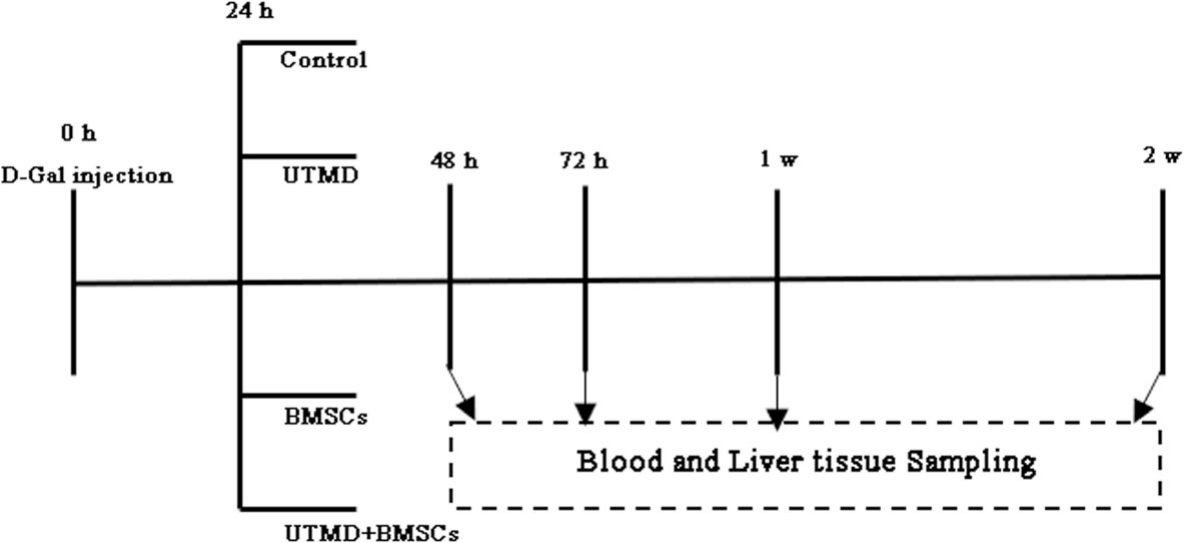
The experimental design and the time course of the interventions

After validation of the ALI model and UTMD parameters, the formal treatment experiment was initiated. Briefly, 48 ALI rats were divided into four groups: (1) control (ALI rats without treatment), (2) BMSCs transplantation, (3) UTMD treatment, and (4) treatment with UTMD + BMSCs. Approximately 2 × 10^6^ BMSCs in 2 mL of PBS were slowly injected into the rats in group 2 via the tail vein at 24 h after ALI was established. The rats in groups 3 and 4 received 1.5 W/cm^2^ ultrasound radiation and 300 μL of microbubbles injected through tail vein; then, the transplantation of BMSCs was performed for group 4. The protocols of UTMD we applied were the same as those described above. The schematic overview of the experimental procedure is illustrated in Fig. 1.

The homing of BMSCs in the injured livers at 48 h after treatment with BMSCs and/or UTMD was analyzed. The recovery of liver function and histology was monitored at days 2, 3, 7, and 14.

### Enzyme-linked immunosorbent assay (ELISA)

The hepatic expression of TNF-α in rats exposed to different ultrasound intensities was analyzed to determine the safety of UTMD. Also, the hepatic expression of monocyte chemotactic protein 1 (MCP-1) in the four groups was analyzed. Both markers were determined in triplicate with ELISA (R&D Systems, Minneapolis, MN, USA).

### Serum biochemical markers

Blood samples were obtained from rats at days 2, 3, 7, and 14 after treatment. The kinetic changes in serum alanine aminotransferase (ALT), aspartate aminotransferase (AST), alkaline phosphatase (ALP), and total bilirubin (TBIL) were determined using assay kits (Nan Jing Jian Cheng, Nan Jing, China).

### Detection of the homing efficacy of BMSCs

To compare the homing efficiency of BMSCs among the different groups, three rats in each group were sacrificed at 48 h after the injection of BMSCs. Liver tissues were frozen and embedded in Optimum Cutting Temperature reagent (Sakura Finetek, Tokyo, Japan). The frozen liver tissues were cut into 8-μm sections to detect the labeled MSCs after the nuclei were stained with 2-(4-amidinophenyl)-6-indolecarbamidine (DAPI). The number of GFP-positive BMSCs in the liver sections was counted under a fluorescence microscope.

After intravenous injection of BMSCs, the levels of the GFP gene were evaluated by quantitative reverse transcription–polymerase chain reaction (qRT-PCR). Taken fluorescent staining and mRNA together, we confirmed the homing rate of BMSCs in liver sections.

### Western blotting

The hepatic expression of four chemokines involved in the migration of BMSCs, including SDF-1, intercellular cell adhesion molecule (ICAM-1), vascular cell adhesion molecule 1 (VCAM-1), and hepatocyte growth factor (HGF), was detected using western blotting. The total protein of the liver tissue was released after lysis with 1 mL of radioimmunoprecipitation assay buffer (Beyotime Biotechnology, Nanjing, China) and 10 μL of phenylmethanesulfonyl fluoride (Beyotime Biotechnology, Nanjing, China) for 30 min on ice. After centrifugation, the supernatants were collected and the protein concentrations were measured by a bicinchoninic acid protein assay kit (Beyotime Biotechnology, Nanjing, China). Equal amounts of protein samples were separated on a sodium dodecyl sulfate–polyacrylamide gel (Beyotime Biotechnology, Nanjing, China) by electrophoresis and then transferred onto a polyvinylidene fluoride membrane (Millipore, Billerica, MA, USA). The blotted membrane was blocked with 5% nonfat powered milk dissolved in tris-buffered saline containing Tween 20 (Sangon Biotech, Shanghai, China) for 1 h and incubated overnight with the following antibodies: anti-SDF-1 (1:1000, Abcam, Cambridge, MA, USA), anti-ICAM-1 (1:1000, Proteintech, Shanghai, China), anti-VCAM-1 (1:1000, Abcam, Cambridge, MA, USA), anti-GAPDH (1:1000, Beyotime, Nanjing, China), and anti-HGF (1:1000, Cell Signaling Technology, Danvers, MA, USA). The membrane was exposed to the horseradish peroxidase (HRP)-conjugated secondary antibody goat anti-rabbit/anti-mouse IgG (1:2000, Abcam, Cambridge, MA, USA) for 1 h at room temperature. Finally, the targeted bands were visualized with enhanced chemiluminescence reagents (Millipore, Billerica, MA, USA), quantified using Image Pro Plus software, and normalized against the internal control GAPDH.

### qRT-PCR

In order to validate the changes in the levels of the expressed cytokines, qRT-PCR was used for mRNA detection of GFP, SDF-1, ICAM-1, VCAM-1, HGF, and MCP-1. The total RNA was extracted from frozen liver tissues with Trizol reagent (Invitrogen, Carlsbad, CA, USA), according to the manufacturer’s instructions. The concentration of RNA was confirmed by Nano Drop 2000 spectrophotometry (Thermo Fisher, Wilmington, DE, USA). The synthesis of the first-strand cDNA template was carried out with a Prime Script RT Master Mix Kit (Takara, Dalian, Liaoning, China) using 500 ng of total RNA in a 10-μL reaction system. qRT-PCR was performed using a Quant Studio 6 Flex Real-Time PCR Detection system (Thermo Fisher, Wilmington, DE, USA) with a SYBR Premix Ex Taq RT-PCR Kit (Takara, Dalian, Liaoning, China). The forward and reverse primers of the target gene are listed in Table 1 (generated by Sangon Biotech, Shanghai, China). The relative target gene expression was calculated using the 2^−ΔΔCt^ method. In addition, the expression level of GAPDH served as the internal reference.

**Table 1:** Sequences of primers used for qRT-PCR

Gene	Forward sequence	Reverse sequence
GFP	CGACCACTACCAGCAGAACA	GAACTCCAGCAGGACCATGT
MCP-1	CCAATGAGTCGGCTGGAGAA	GTGCTTGAGGTGGTTGTGGAA
ICAM-1	TGTCGGTGCTCAGGTATCCATCC	TTCGCAAGAGGAAGAGCAGTTCAC
VCAM-1	TTTGCAAGAAAAGCCAACATGAAAG	TCTCCAACAGTTCAGACGTTAGC
SDF-1	GCTCTGCATCAGTGACGGTA	TGCACACTTGTCTGTTGTTGC
HGF	CAATCCAGAGGTACGCTACGA	TGCCTGATTCTGTGTGATCC
TNF-α	TGATCCGAGATGTGGAACTG	CGAGCAGGAATGAGAAGAGG
GAPDH	TGCACCACCAACTGCTTAG	GATGCAGGGATGATGTTC

### Histological analysis

The livers of the experimental groups were collected at 48, 72, 168, and 336 h post treatment. The specimens were fixed in 10% *neutral* buffered *formalin*, embedded in paraffin, and cut into 5-μm sections. After hematoxylin–eosin (H&E) staining, liver pathological findings and images were assessed under a microscope (Leitz Aristoplan, Wetzlar, Germany). For immunohistochemical analysis, the liver sections at days 7 and 14 were stained with the anti-α-smooth muscle actin (α-SMA) antibody (1:200, Abcam, Cambridge, MA, USA), and then, the signals were visualized with the substrate dimethylaminoazobenzene (DAB) (Vector Laboratories, Burlingame, CA, USA). The α-SMA expression level was quantified in six to eight random microscopic fields using Image Pro Plus software.

### TUNEL assay

After a 2-week treatment, the apoptosis rate of hepatocytes in the four groups was assessed by in situ detection of DNA fragmentation using a TdT-mediated dUTP nick end-labeling (TUNEL) assay kit (Roche, Mannheim, Germany). Briefly, the endogenous peroxidase activity in paraffin sections was blocked with 2% hydrogen peroxide in PBS, then incubated with TUNEL reaction solution, and directly stained with freshly prepared 0.05% DAB solution for 3–6 min. The green apoptosis-positive cells were counted in three fields for each section under a fluorescence microscope (Leitz Aristoplan, Wetzlar, Germany).

### Immunohistochemical analysis

Proliferating cell nuclear antigen (PCNA) immunofluorescence was employed to identify the regeneration of hepatocytes using the specific antibody anti-PCNA (1:50, Abcam, Cambridge, MA, USA) and an ABC staining kit (Vector Laboratories, Burlingame, CA, USA). The number of red PCNA-positive cells was counted and averaged in each sample by three pathologists blinded to the animal experiments.

### Statistical analysis

All continuous variables were reported as means ± standard deviation for each group. Student’s *t* test and multiple factor analysis of variance were used to compare differences between groups. A *p* value < 0.05 was considered statistically significant. All data were processed using the statistical analysis software SPSS (v. 20.0 Armonk, NY, USA).

## Results

### Culture and identification of rat BMSCs

**Fig. 2:**
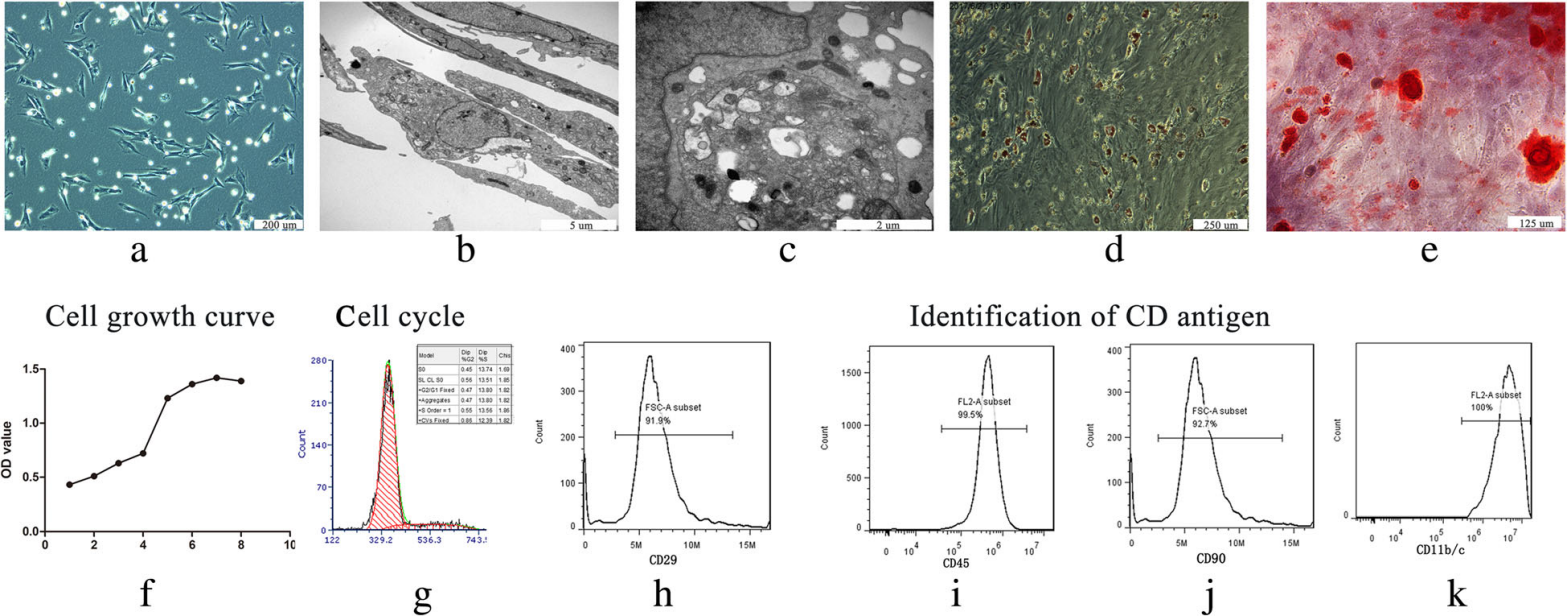
Characterization of radifferentiation characteristicst BMSCs. Morphological characteristics of BMSCs under microscope (**a**) and TEM (**b**, **c**). The adipogenic (**d**) and osteogenic induction (**e**) demonstrated good differentiation features. The cell cycle showed that most cells were at G0/G1 (**g**). Cell counting kit-8 staining demonstrated the proliferation capability of BMSCs (**f**). Results of  the CD phenotypic antigen identification were: CD29+ (**h**), CD45- (*i*), CD90+ (**j**), CD11b/c - (**k**)

The freshly isolated BMSCs were spherical in morphology. After 8 to 13 days of culture, the BMSCs reached 80% confluence and were passaged. The passaged cells became more uniform and appeared as a long-spindle shape (Fig. 2a). The TEM images showed that they contained abundant mitochondria, ribosomes, endoplasmic reticulum, lysosomes, and secretory vesicles in the cytoplasm of BMSCs (Fig. 2b, c).

After culturing with osteogenic induction medium for approximately 14 days, the cells were fixed with paraformaldehyde. The cells incubated with adipogenic medium formed visible lipid droplets (Fig. 2d). Similarly, calcium nodules stained with Alizarin Red were easily observed under a fluorescence microscope (Fig. 2e).

The CCK-8 data showed that the growth curve of BMSCs presented an “S” type growth (Fig. 2f). The cell cycle results demonstrated that the majority of cells were in the G0 + G1 phase (69.64%) (Fig. 2g). Both of these findings indicated that the BMSCs possessed high proliferation characteristics.

The expression of the CD molecule was detected by flow cytometry. The data showed a high level of CD29 and CD90 expression, but the absence of CD11b/c and CD45 expression. The percentages were as follows: CD29, 91.9%; CD90, 92.7%; CD11b/c, 0.01%; and CD45, 0.5% (Fig. 2h–k).

### Verification of lentivirus-transduced BMSCs

**Fig. 3:**
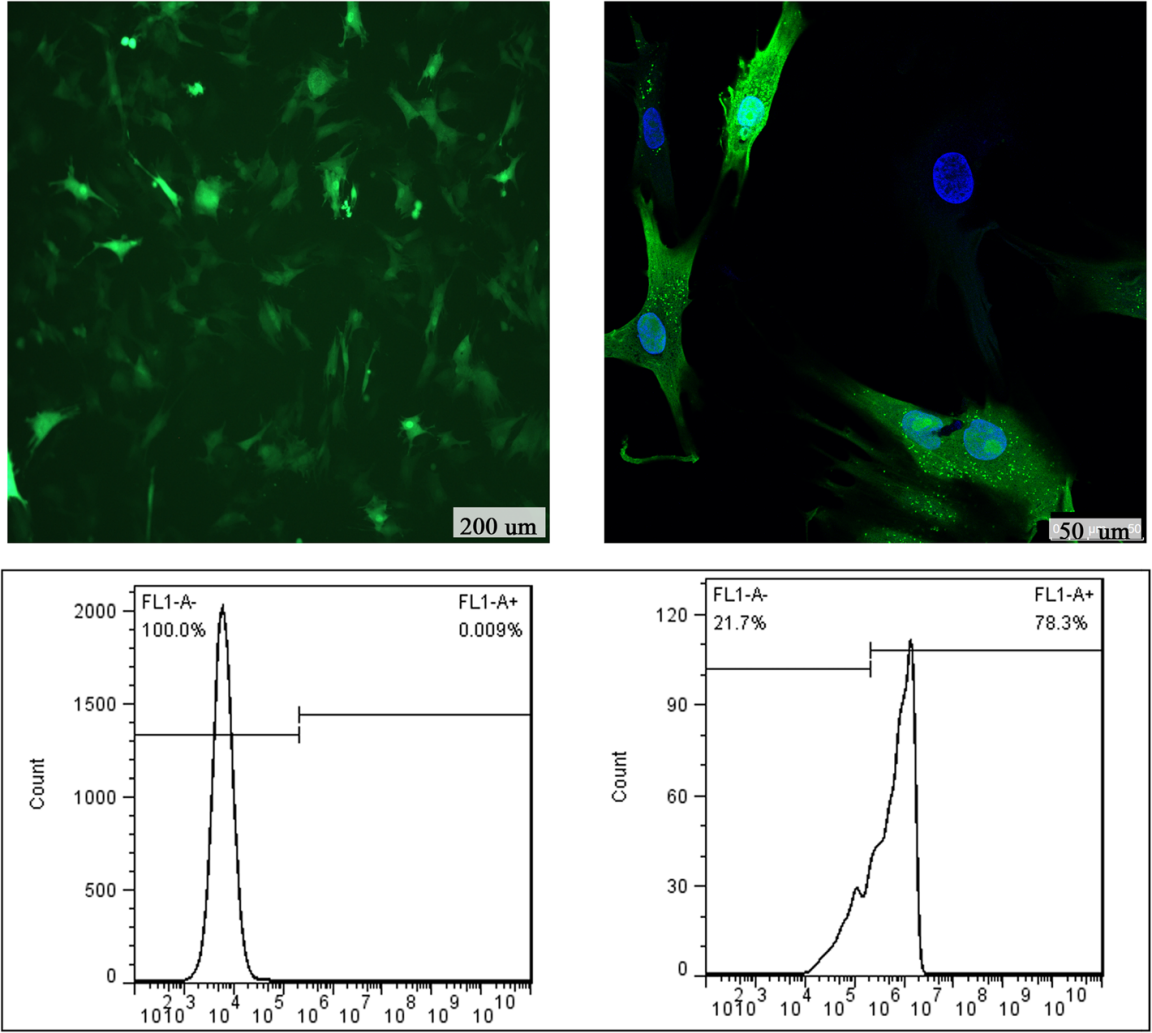
BMSCs transduced with lentivirus expressing GFP. Cells were observed under a fluorescence microscope (top left, scale bar = 200 μm), a confocal microscope (top right, scale bar = 50 μm), and analyzed by flow cytometry (bottom)

The lentivirus-traduced BMSCs showed bright green fluorescence under a fluorescence microscope (Fig. 3). The positive transduction rate was approximately 78.3%. The transduced BMSCs maintained characteristics of stem cells while stably expressing green fluorescence after the passage.

### Evaluation of the ALI model

**Fig. 4:**
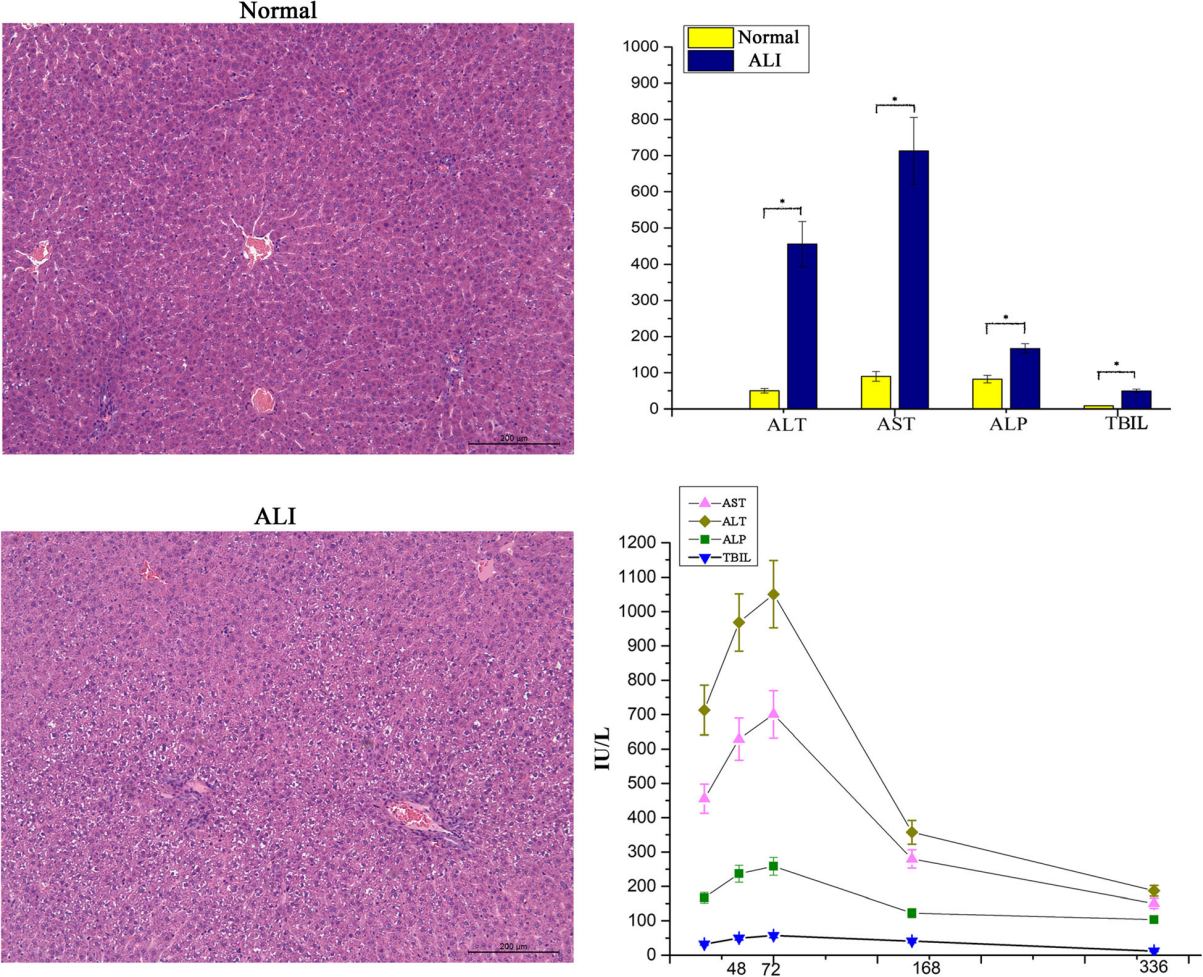
Confirmation of ALI in rats (*n* = 3 per group). Multiple diffused necrosis was seen after the injection of D-Gal (H&E staining, magnification × 100, scale bar = 200 μm). AST, ALT, ALP, and TBIL were significantly higher in the model group (*p ≤ 0.05)

D-Gal has been widely used for the induction of ALI in animal models [17, 18]. Two of 24 rats died after the injection of D-Gal. ALT, AST, ALP, and TBIL became significantly elevated in the model group (ALT 455.36 ± 62.54 vs. 50 ± 6.12 U/L; AST 713.11 ± 92.54 vs. 90 ± 13.42 U/L; ALP 167.01 ± 13.22 vs. 72.36 ± 8.17; TBIL 8.55 ± 0.62 vs. 49.4 ± 5.16 U/L (*p* < 0.05)) (Fig. 4). H&E staining of the ALI liver sections showed hepatocyte cytoplasm edema, necrosis, and inflammatory cell infiltration (Fig. 4).

The serum biochemical indicators for ALI rats were detected for 14 days (Fig. 4). ALT, AST, ALP, and TBIL reached the peak at 72 h after the injection of D-Gal. Then, the serum markers gradually decreased. The liver enzymes in the ALI rats declined and remained at a slightly higher level on day 10.

### Optimization of ultrasound parameters

**Fig. 5:**
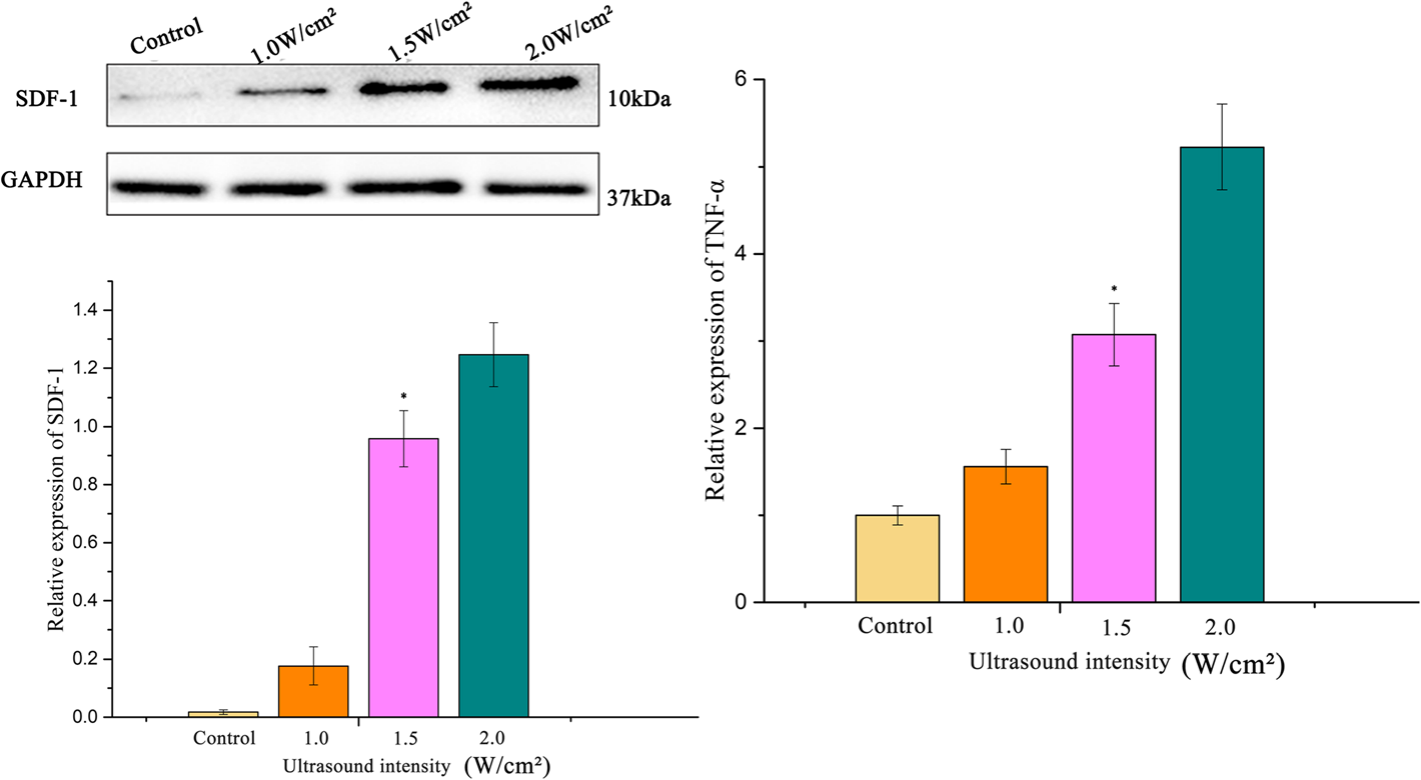
Impact of UTMD on ALI rats (*n* = 3 per group). Western blot analysis indicated that UTMD upregulated SDF-1 expression. The cytokine levels indicated that the liver injury was significantly aggravated under an ultrasound power of 2.0 W/cm^2^ (*p ≤ 0.05)

The expression of SDF-1 and TNF-α was elevated in rats that received irradiation with increased ultrasound intensities (Fig. 5). Interestingly, compared with 1.5 W/cm^2^ UTMD, the level of TNF-α in the rats exposed to 2.0 W/cm^2^ UTMD was significantly increased (1.1 vs. 1.3). However, the SDF-1 expression level was only modestly elevated (3.0 vs. 5.2).

The principle to select optimal ultrasound conditions was to maximize the UTMD power without causing tissue damage. Therefore, an intensity at 1.5 W/cm^2^ UTMD was found to be optimal for the transplantation of BMSCs, while minimizing liver injury.

### Homing efficiency of BMSCs

**Fig. 6:**
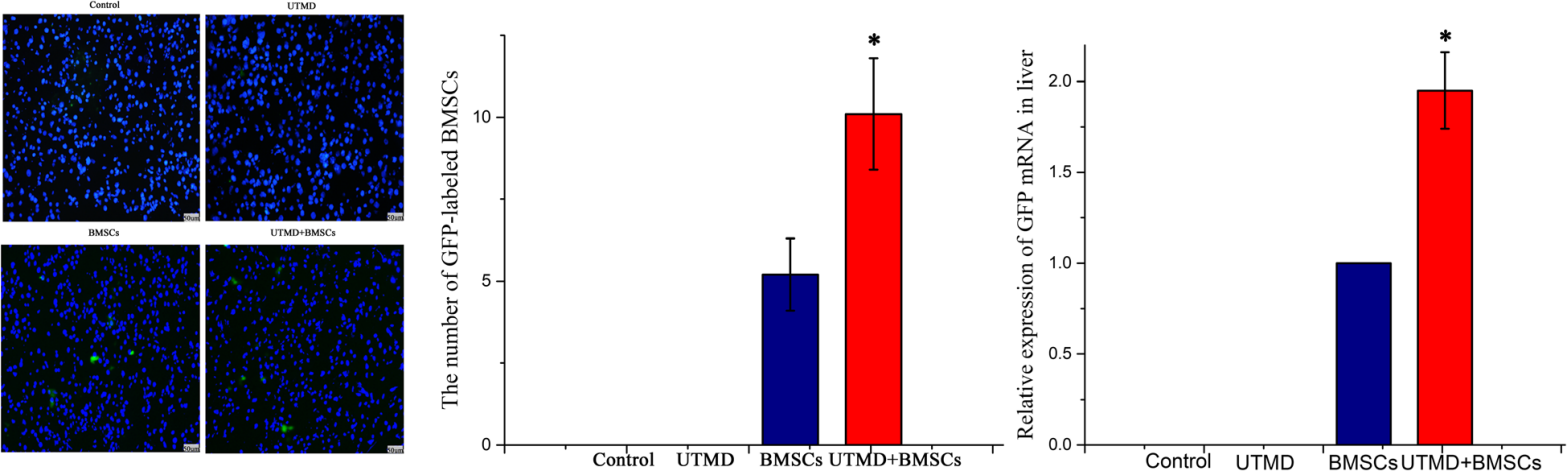
Homing efficiency of BMSCs in the different groups (scale bar = 50 μm) (*p ≤ 0.05)

To evaluate the migration efficiency of BMSCs in combination with UTMD, liver sections in the four groups from day 2 post treatment were stained with DAPI. Six microscopic fields were randomly selected to count the EGFP-positive cells, which were significantly higher in the UTMD + BMSCs group (9.8 ± 2.3) than in the other groups (0, 0, 5.2 ± 1.1) (Fig. 6).

To increase the sensitivity, we selected qRT-PCR for the detection of GFP-positive transplanted cells in the liver with a more quantitative method. The results were consistent with those under light microscopy (Fig. 6). We found an approximately twofold increase in homing of GFP-labeled BMSCs in the liver in the UTMD + BMSCs group, compared with the BMSCs group.

### Effect of BMSCs on recovery of liver function and histology

**Fig. 7:**
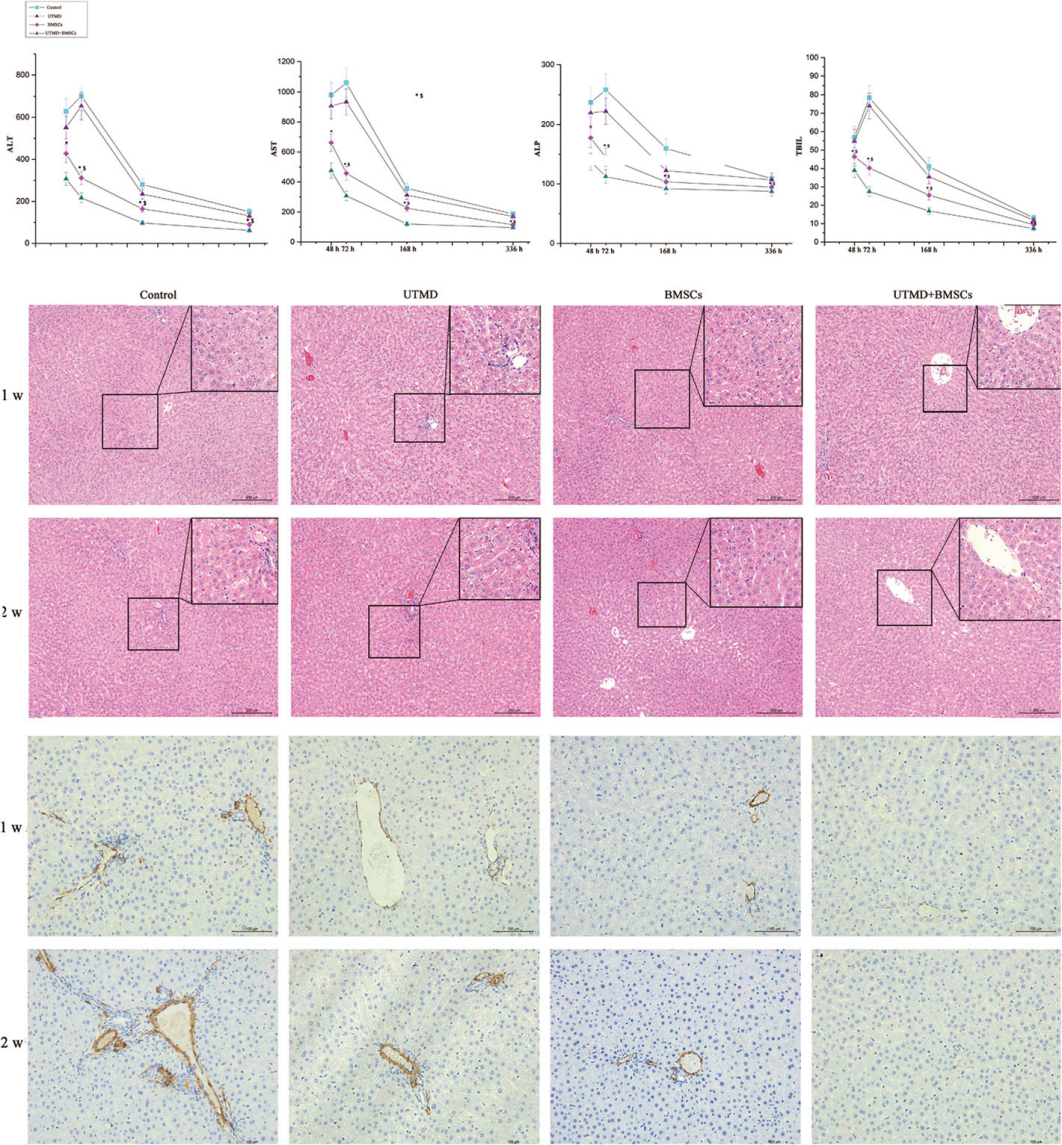
The rats with ALI induced by D-Gal and treated with transplanted BMSCs in combination with UTMD had significantly improved liver function, inflammation, and survival. The kinetic levels of ALT, AST, ALP, and TBIL (top). Representative images of H&E staining (middle, scale bar = 200 μm) and α-SMA immunohistochemistry staining (bottom, scale bar = 100 μm) demonstrated significantly decreased liver injury in the UTMD + BMSCs group (*p < 0.01, compare with other groups, $*p* < 0.01, compare with intragroup at different time points)

The serum levels of ALT, AST, ALP, and TBIL in the UTMD + BMSCs group were significantly lower than those in the BMSCs group at days 3, 4, and 7 (Fig. 7). At 72 h after BMSCs transplantation, there was a significant decline in the levels of serum biological markers, and no significant differences were noted at day 14 after treatment.

In the control group at 48 h, we observed that the hepatic cord architecture collapsed and was interwoven with necrotic hepatocytes and infiltrated neutrophils; at 72 h, massive patchy necrotic hepatocytes and cell debris developed. After BMSCs transplantation, H&E staining of the liver showed mild hepatocyte degeneration and inflammatory cell infiltration (Fig. 7). There were no significant differences between the UTMD + BMSCs group and the BMSCs group. Reduced sinusoidal congestion, inflammatory cell infiltration, and trabecular fragmentation were revealed after BMSCs transplantation. Furthermore, significant differences in pathological changes were noted between the UTMD + BMSCs group and the BMSCs group.

Liver tissues in the control group were congested with cell necrosis and minor bile duct proliferation after 168 h. In the BMSCs group, there was only minor hepatocyte inflammation and necrosis. The hepatic lobular structure gradually returned to normal, though focal inflammatory cells remained seen in the UTMD + BMSCs group. Significant differences in morphological changes were documented between these two groups. Liver tissues in the control group showed fibrous hyperplasia in the hepatic lobules as well as residual necrosis with less prominent inflammatory cells at 14 days after treatment. In the UTMD + BMSCs group and the BMSCs group, the hepatic lobular structures became almost normal without a significant difference between the UTMD + BMSCs group and the BMSCs group.

Additionally, the number of α-SMA-positive cells was significantly lower in the liver tissues of the UMTD + BMSCs group compared to the BMSCs group at days 7 and 14 (Fig. 7).

### Hepatic levels of SDF-1, VCAM-1, ICAM-1, MCP-1, and HGF

**Fig. 8:**
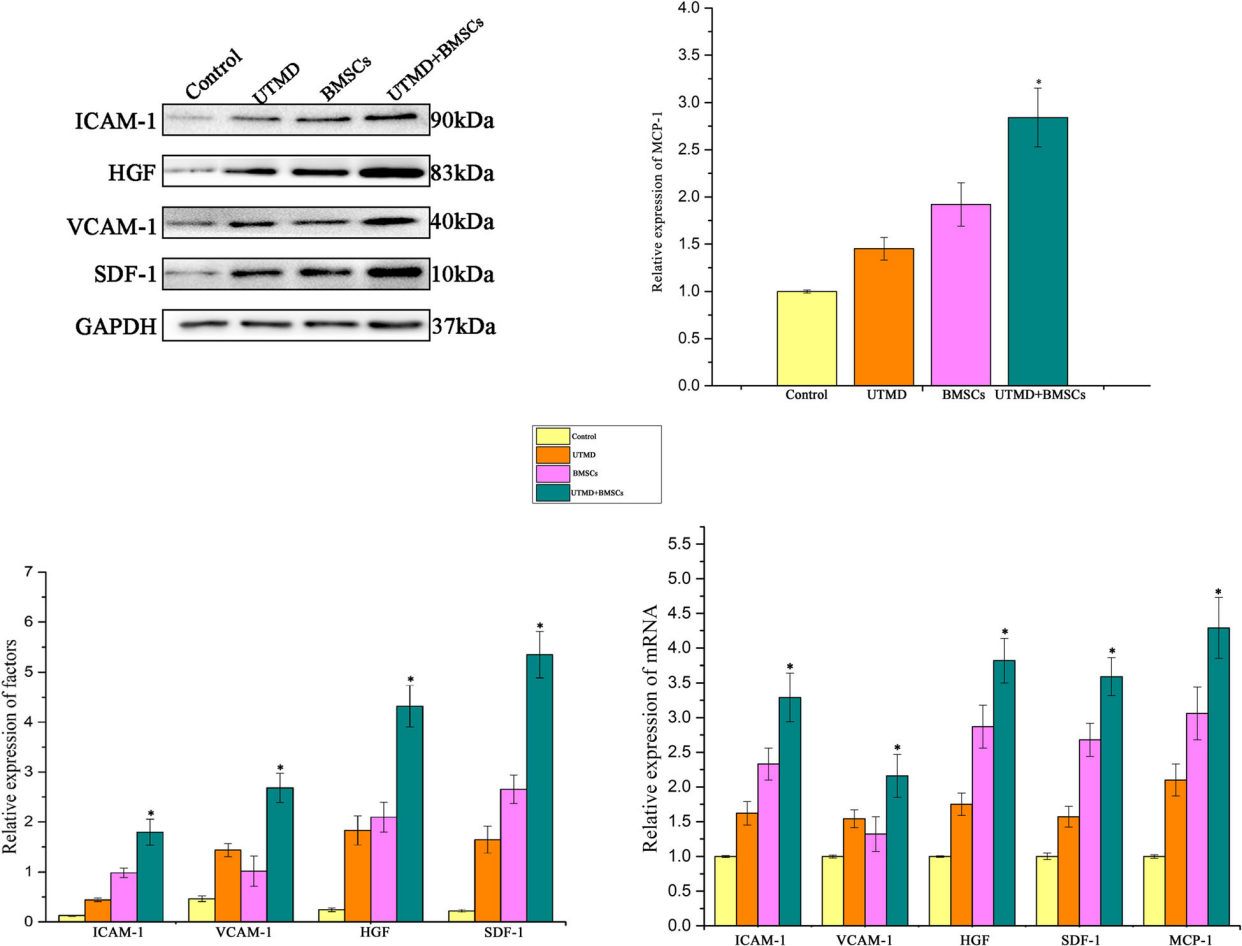
The relative protein and mRNA levels of hepatic SDF-1, ICAM-1, VCAM-1, and HGF (*n* = 3 per group) to that of GAPDH (internal control). The hepatic MCP-1 level was detected using ELISA analysis (*p ≤ 0.05)

Hepatic expression of SDF-1, VCAM-1, ICAM-1, and HGF was detected by western blotting at 3 days after treatment (Fig. 8). The protein expression of MCP-1, an important factor involved in the migration of BMSCs, was analyzed by ELISA. The variation tendencies of SDF-1, VCAM-1, and MCP-1 were approximately the same. The hepatic expression levels of ICAM-1, HGF and MCP-1 were 0.13 ± 0.011, 0.44 ± 0.035, 0.98 ± 0.12, and 1.79 ± 0.42; 0.24 ± 0.017, 1.83 ± 0.29, 2.11 ± 0.31, and 4.32 ± 0.66; 1.02 ± 0.015, 1.45 ± 0.12, 1.92 ± 0.23, and 2.83 ± 0.31 (*p* < 0.01) in the control, UTMD, BMSCs, and UTMD + BMSCs groups, respectively. The results showed that SDF-1, a major mediator involved in the homing of BMSCs in the injured liver, was markedly upregulated after the application of UTMD. The VCAM-1 protein levels were 0.23 ± 0.0125, 1.64 ± 0.41, 1.03 ± 0.27, and 2.68 ± 0.45 (*p* < 0.01) in the control, UTMD, BMSCs, and UTMD + BMSCs groups, respectively. The SDF-1 protein levels were 0.20 ± 0.125, 1.44 ± 0.21, 2.67 ± 0.47, and 5.35 ± 0.78 (*p* < 0.01), respectively. The HGF protein is an indicator for hepatocyte recovery, and its relative expression level was significantly higher in the UTMD + BMSCs group compared to the other groups. The expression of apoptotic protein Bcl-2 and Bax in targeted liver 72 h after treatment were also detected using Western Blot. Results demonstrated that ratio of Bcl-2/Bax was the highest in UTMD+BMSCs group (seen in Additional file 1).

### Hepatic mRNA levels of SDF-1, VCAM-1, ICAM-1, HGF, and MCP-1

The hepatic mRNA levels of SDF-1, VCAM-1, ICAM-1, MCP-1, and HGF at 72 h after treatment were measured by qRT-PCR (Fig. 8), and the relative mRNA levels were consistent with the corresponding protein levels.

### Immunohistochemical staining of PCNA

**Fig. 9:**
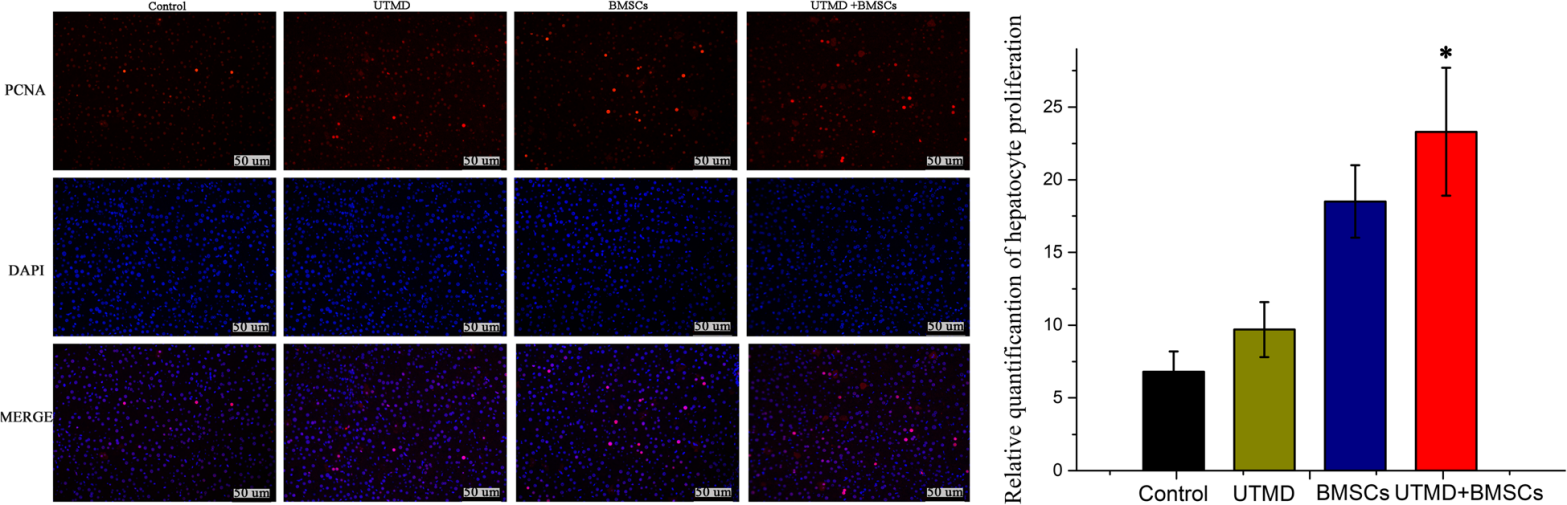
Immunohistochemical staining of PCNA in the four groups (n = 3 per group). The cells with positive proliferation were identified with red fluorescence (scale bar = 50 μm) (*p ≤ 0.05)

PCNA-positive hepatocytes were measured at day 14 by immunofluorescence (Fig. 9). The PCNA staining intensity in the UTMD + BMSCs group and the BMSCs group was stronger than that of the control and UTMD groups. Moreover, there were markedly differences in PCNA staining between the UTMD + BMSCs group and the BMSCs group.

### TUNEL assay

**Fig. 10:**
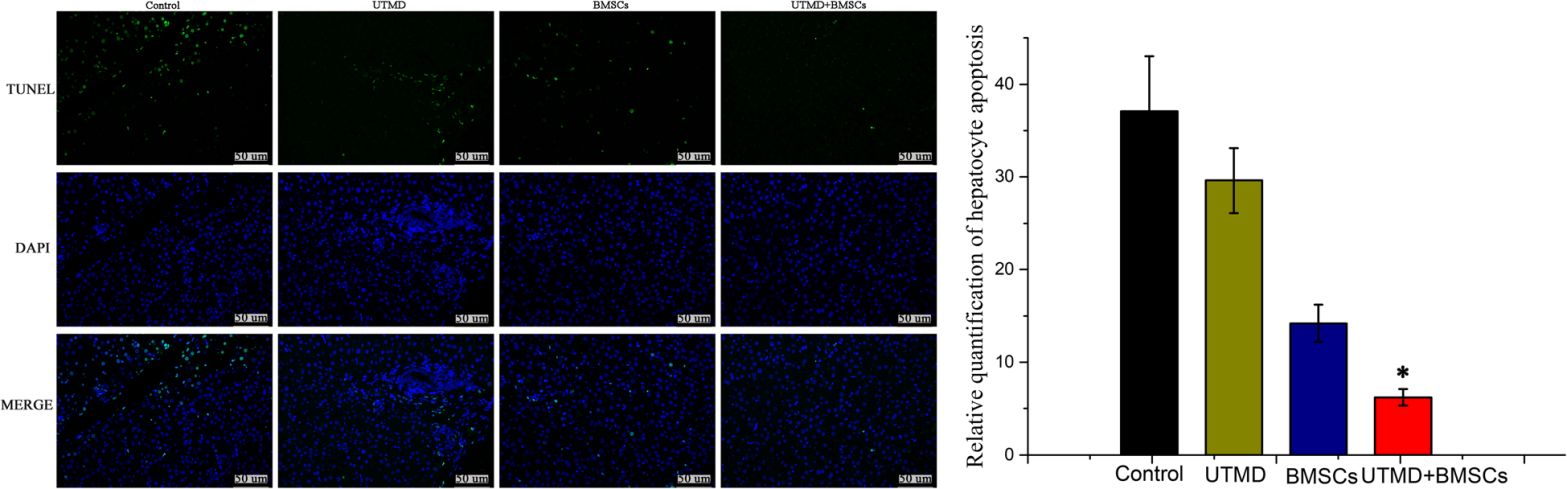
TUNEL assay of apoptosis in the four groups (*n* = 3 per group). The positive apoptotic cells were marked with green fluorescence (scale bar = 50 μm) (*p ≤ 0.05)

Hepatocyte apoptosis was detected by TUNEL staining (Fig. 10), and the number of apoptotic hepatocytes was significantly higher in the control group and the UTMD group at day 7. The positive apoptotic cells were stained with green fluorescence, showed a blue nucleus, and displayed a round shape. The number of apoptotic cells became significantly lower in the UTMD + BMSCs group and the BMSCs group at 168 h following BMSCs transplantation (13 ± 2.3 vs. 6.2 ± 0.9) (*p* < 0.05).

## Discussion

D-Gal is a well-known toxicant that induces hepatotoxicity and specifically interferes with uridine diphosphate of hepatocytes [19, 20]. In this study, a single dose of D-Gal (1.5 g/kg) was used to induce hepatotoxicity, which generated severe liver injury at 24 h after drug administration.

Currently, stem cell implantation, which provides an additional source of stem cells, can be used for regenerating hepatocytes to repair an injured liver, including acute and chronic liver failure [21]. Previous studies have reported that the engraftment of BMSCs for the treatment of liver injury is safe and feasible, but the therapeutic efficacy is limited by the lower liver recruitment of BMSCs upon transplantation [22].

Recent studies have supported that UTMD treatment can increase the number of endothelial cells and interstitial capillary permeability as well as facilitate drug and gene delivery to target sites [23, 24]. In addition, animal studies have shown that UTMD causes microvascular rupture in the target organs, resulting in the upregulation of certain cytokines, accompanied by the increased migration of BMSCs into the heart and kidney [25–28].

Our group has previously reported that the UTMD treatment markedly enhances the efficiency of transfection of BMSCs with the gene encoding HGF [29]. In this study, we explored the approach that combined BMSCs transplantation with the prior UTMD procedure in treating injured rat livers. We aimed to investigate whether an optimized acoustic cavitation would improve the homing of BMSCs through providing a suitable local microenvironment [30, 31]. This study represents progress from our previous in vitro experiment and a first attempt to perform in vivo evaluation in the setting of ALI.

We optimized the parameters required for achieving a high efficiency of UTMD without inducing significant liver injury, and then, the selected conditions were applied for the UTMD procedure before the transplantation of BMSCs in ALI rats. The results showed that the number of liver homing BMSCs was significantly higher in the UTMD + BMSCs group compared to the BMSCs group at 48 h after cell transplantation. Our findings were consistent with previous studies, in which the UTMD procedure was conducted before the injection of BMSCs, and the combination provided a beneficial impact to the engraftment of cells [32–34].

To better understand the underlying mechanism, we investigated the biological effects of UTMD on the targeted livers. Both western blotting and qRT-PCR showed that the expression of adhesion molecules and cytokines was significantly higher in the UMTD + BMSCs group. We analyzed in detail the expression of SDF-1, VCAM-1, ICAM-1, and MCP-1, four important molecules involved in the liver homing of BMSCs [35–37]. The detected mRNA levels correlated well with the corresponding protein levels.

Our results showed that upregulation of the SDF-1 level after UTMD pretreatment was associated with the increased homing efficiency of BMSCs into the liver. The increased ICAM-1 and VCAM-1 expression suggested that UTMD may have enhanced the intercellular and interstitial capillary permeability, which also facilitated the efficient migration of BMSCs. One unexpected finding was that the VCAM-1 expression level was not in parallel with the ICAM-1 expression level. We reasoned that the VCAM-1 gene expression may react differently to UTMD intervention. It did not appear that BMSCs affect the secretion of VCAM-1 through the paracrine mode. Similarly, UTMD was associated with the enhanced expression of MCP-1, which is an important adhesion molecule closely related to the homing of BMSCs. Our results also demonstrated that the UTMD procedure under the optimized conditions may keep liver side effects to a minimal level [38, 39].

We found that the UTMD + BMSCs treatment significantly improved the recovery from severe liver injury as demonstrated by liver histology, immunofluorescence of PCNA, TUNEL staining, and serum biochemical markers. HGF may participate in liver remodeling and regenerative processes; however, we did not research this in depth. The expression of HGF was significantly improved at days 7 and 14 after the injection of BMSCs in the UTMD + BMSCs group, compared to the BMSCs group (shown in Additional file 1). These results suggest that the mechanical function of UTMD could positively impact the process of hepatocyte regeneration and repair.

Systematic detection of the following survival and fate of BMSCs in the injured liver was not performed in our study. This focus will be addressed in our future studies. Also, we will carry out further in-depth studies in our future work, such as whether the fate of transplanted BMSCs could be affected by the application of UTMD.

## Conclusion

In this study, we optimized the UTMD ultrasound parameters that efficiently induced the microenvironment to make it favorable for cell migration in this study. Then, we demonstrated that a UTMD procedure prior to the intravenous infusion of BMSCs facilitated the liver homing of BMSCs. The enhanced liver homing efficiency by UTMD was likely mediated through the upregulated expression of adhesion molecules and cytokines. The enhanced homing and migration of BMSCs led to a significantly improved recovery of histology and serum biochemical markers in severely injured livers. These findings provide a preliminary proof-of-concept that a combination of UTMD with transplanted BMSCs is feasible and efficient, presenting therapeutic benefits in treating severely injured livers in a rat model.

## Additional file

Additional file 1: **Figure S1.** Expression of the protein Bcl-2 and Bax in rats’ liver 2 weeks after UTMD/BMSCs treatment.Furthermore, the level of HGF was also calculated to assess the recovery of liver 2 weeks after experiment. (DOCX 134 kb)

## Abbreviations

CD: Cluster of differentiation
DAPI: 2-(4-Amidinophenyl)-6-indolecarbamidine
ECL: Enhanced chemiluminescence
EGFP: Enhanced green fluorescent protein
FDA: Food and Drug Administration
MFI: Mean fluorescence intensity
MOI: Multiplicity of infection
OCT: Optimum cutting temperature
PBS: Phosphate-buffered saline
PMSF: Phenylmethanesulfonyl fluoride
RIPA: Protein lysis buffer radio immunoprecipitation assay
TBST: Tris-buffered saline with Tween20

## References

[1] Thawley V (2017). Acute liver injury and failure. Vet Clin North Am Small Anim Pract.

[2] Londono MC, Rimola A, O’Grady J (2013). Immunosuppression minimization vs. complete drug withdrawal in liver transplantation. J Hepatology.

[3] Zhang Z, Wang FS (2013). Stem cell therapies for liver failure and cirrhosis. Hepatology.

[4] Christ B, Brückner S, Winkler S (2015). The therapeutic promise of mesenchymal stem cells for liver restoration. Trends Mol Med.

[5] Volarevic V, Nurkovic J, Arsenijevic N (2014). Concise review: therapeutic potential of mesenchymal stem cells for the treatment of acute liver failure and cirrhosis. Stem Cells.

[6] Shiratsuki S, Terai S, Murata Y (2016). Enhanced survival of mice infused with bone marrow-derived as compared with adipose-derived mesenchymal stem cells. Hepatol Res Official J Jpn Soc Hepatol.

[7] Meirelles LDS, Fontes AM, Covas DT (2009). Mechanisms involved in the therapeutic properties of mesenchymal stem cells. Cytokine Growth Factor Rev.

[8] Martin-Rendon E, Brunskill S, Doree C J, et al. Stem cell treatment for acute myocardial infarction[M]// The Cochrane Library. John Wiley & Sons, Ltd, 2007:CD006536. 10.1002/14651858.CD006536.pub2 18843721

[9] Price RJ, Kaul S (2002). Contrast ultrasound targeted drug and gene delivery: an update on a new therapeutic modality. J Cardiovasc Pharmacol Ther.

[10] Khokhlova TD, Haider Y, Hwang JH. Therapeutic potential of ultrasound microbubbles in gastrointestinal oncology: recent advances and future prospects. Therap Adv Gastroenterol. 2015;8(6):384-94. 10.1177/1756283X15592584 PMC4622285 26557894

[11] Delalande A, Postema M, Mignet N, Midoux P, Pichon C (2012). Ultrasound and microbubble-assisted gene delivery: recent advances and ongoing challenges. Ther Deliv.

[12] Imada T, Tatsumi T, Mori Y (2005). Targeted delivery of bone marrow mononuclear cells by ultrasound destruction of microbubbles induces both angiogenesis and arteriogenesis response. Arterioscler Thromb Vasc Biol.

[13] Wang G, Zhang Q, Zhuo Z (2016). Enhanced homing of CXCR-4 modified bone marrow-derived mesenchymal stem cells to acute kidney injury tissues by micro-bubble-mediated ultrasound exposure. Ultrasound Med Biol.

[14] Maes M, Vinken M, Jaeschke H (2016). Experimental models of hepatotoxicity related to acute liver failure. Toxicol Appl Pharmacol.

[15] Drucker C, Rabe B, Chalaris A (2009). Interleukin-6 trans-signaling regulates glycogen consumption after D-galactosamine-induced liver damage. J Interf Cytokine Res.

[16] Éboli LP, Netto AA, Azevedo RA (2016). Evaluating the best time to intervene acute liver failure in rat models induced by d-galactosamine. Acta Cirurgica Brasileira.

[17] Song S, Noble M, Sun S (2012). Efficient microbubble- and ultrasound-mediated plasmid DNA delivery into a specific rat liver lobe via a targeted injection and acoustic exposure using a novel ultrasound system. Mol Pharm.

[18] Yang D, Tan KB, Gao YH (2012). Effects of diagnostic ultrasound-targeted microbubble destruction on permeability of normal liver in rats. Ultrasonics.

[19] Pushpavalli G, Kalaiarasi P, Veeramani C (2010). Effect of chrysin on hepatoprotective and antioxidant status in D-galactosamine-induced hepatitis in rats. Eur J Pharmacol.

[20] Zhang L, Kang W, Lei Y, et al. Granulocyte colony-stimulating factor treatment ameliorates liver injury and improves survival in rats with D-galactosamine-induced acute liver failure. Toxicol Lett. 2011;204(1):92-9. 10.1016/j.toxlet.2011.04.016 21550386

[21] Wang K, Li Y, Zhu T (2017). Overexpression of c-Met in bone marrow mesenchymal stem cells improves their effectiveness in homing and repair of acute liver failure. Stem Cell Res Ther.

[22] Vassilopoulos G, Wang PR, Russell DW (2003). Transplanted bone marrow regenerates liver by cell fusion. Nature.

[23] Kiessling F, Fokong S, Koczera P, Lederle W, Lammers T (2012). Ultrasound microbubbles for molecular diagnosis, therapy, and theranostics. Nucl Med.

[24] Collis J, Manasseh R, Liovic P, Tho P, Ooi A, Petkovic-Duran K, Zhu Y (2010). Cavitation microstreaming and stress fields created by microbubbles. Ultrasonics.

[25] Enomoto S, Yoshiyama M, Omura T (2006). Microbubble destruction with ultrasound augments neovascularisation by bone marrow cell transplantation in rat hind limb ischemia. Heart.

[26] Wu S, Li L, Wang G (2014). Ultrasound-targeted stromal cell-derived factor-1-loaded microbubble destruction promotes mesenchymal stem cell homing to kidneys in diabetic nephropathy rats. Int J Nanomedicine.

[27] Zhong S, Shu S, Wang Z (2012). Enhanced homing of mesenchymal stem cells to the ischemic myocardium by ultrasound-targeted microbubble destruction. Ultrasonics.

[28] Skyba DM, Price RJ, Linka AZ (1998). Direct in vivo visualization of intravascular destruction of microbubbles by ultrasound and its local effects on tissue. Circulation.

[29] Fan L, Yang L, Cai Y (2018). Ultrasound irradiation combined with hepatocyte growth factor accelerate the hepatic differentiation of human bone marrow mesenchymal stem cells. Ultrasound Med Biol.

[30] Karp JM, Leng Teo GS (2009). Mesenchymal stem cell homing: the devil is in the details. Cell Stem Cell.

[31] Greco SJ, Rameshwar P (2008). Microenvironmental considerations in the application of human mesenchymal stem cells in regenerative therapies. Biologics Targets Therapy.

[32] Yi Z, Ye C, Gong W (2013). Kidney-targeted transplantation of mesenchymal stem cells by ultrasound-targeted microbubble destruction promotes kidney repair in diabetic nephropathy rats. Biomed Res Int.

[33] Kokhuis TJA, Skachkov I, Naaijkens BA (2015). Intravital microscopy of localized stem cell delivery using microbubbles and acoustic radiation force.

[34] Li L, Wu S, Li P (2015). Hypoxic preconditioning combined with microbubble-mediated ultrasound effect on MSCs promote SDF-1/CXCR4 expression and its migration ability: an in vitro study. Cell Biochem Biophysics.

[35] Belemabedada F, Uchida S, Martire A (2008). Efficient homing of multipotent adult mesenchymal stem cells depends on FROUNT-mediated clustering of CCR2. Cell Stem Cell.

[36] Togel FE, Westenfelder C (2011). Role of SDF-1 as a regulatory chemokine in renal regeneration after acute kidney injury. Kidney International Supplements.

[37] Kuo TK, Hung S, Chuang C (2008). Stem cell therapy for liver disease: parameters governing the success of using bone marrow mesenchymal stem cells. Gastroenterology.

[38] Prentice P, Cuschieri A, Dholakia K (2005). Membrane disruption by optically controlled microbubble cavitation. Nat Phys.

[39] Marie VFGG, Shi WT, Earl PJ (2012). Monitoring and control of microbubble cavitation in therapeutic ultrasound.

