# Supplementary material for: A combination of ultrasound-targeted microbubble destruction with transplantation of bone marrow mesenchymal stem cells promotes recovery of acute liver injury
Source: Stem Cell Res Ther. 2018 Dec 29;9:356. doi: 10.1186/s13287-018-1098-4 (PMC6311028; doi:10.1186/s13287-018-1098-4)
Supplement: Supplementary file 1 — Figure S1. Expression of the protein Bcl-2 and Bax in rats’ liver 2 weeks after UTMD/BMSCs treatment.Furthermore, the level of HGF was also calculated to assess the recovery of liver 2 weeks after experiment. (DOCX 134 kb) [file 13287_2018_1098_MOESM1_ESM.docx]

**Additional file 1**

We detected the expression of apoptotic protein—Bcl-2 and Bax 72h after treatment. It is believed that the increase of Bcl-2/Bax ratio indicates resistance of cells to apoptosis. Results showed the increase of bcl-2 and decrease of Bax in the four groups (Fig 1.). The ratio of Bcl-2/Bax, which is a key factor that regulates cell death, was the highest in UTMD+BMSCs group. Therefore, we believe that UTMD could promote the anti-apoptosis effect of BMSCs.

Previous lectures have proved that when BMSCs was cultured with HGF, an essential factor involved the regeneration and development of hepatocytes, presented an improved therapeutic effect for liver. Hence, we analyzed the protein level of HGF 2 weeks after experiment. In UTMD+BMSCs group, the expression of HGF was the highest, and there was significantly significance between UTMD+BMSCs group ang other groups (Fig 1.). The result was in consistent with the recovery of liver function and histology.


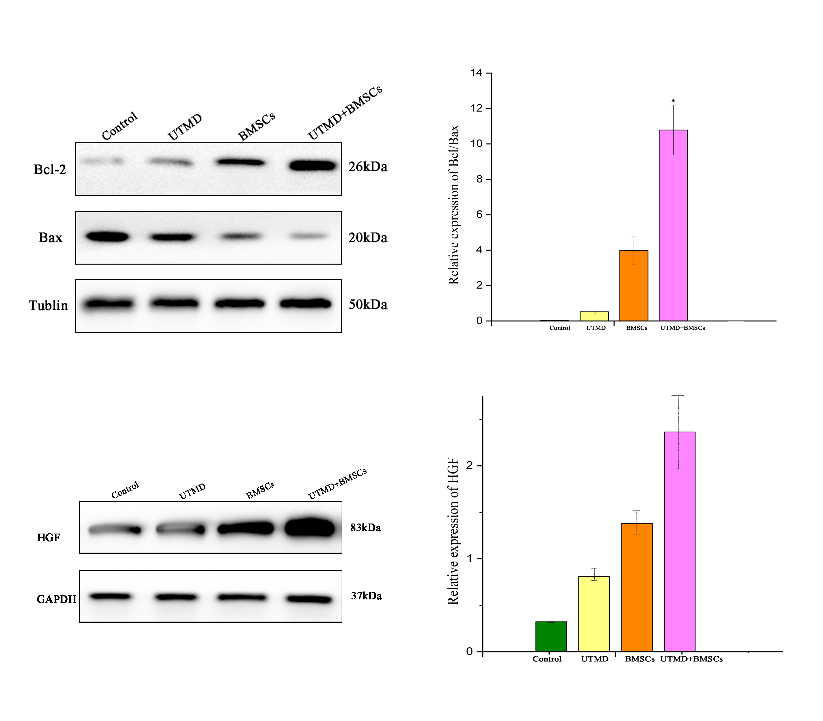


Figure S1. Western blot of Bcl-2,Bax and HGF in the four groups. There were statistically differences between UTMD+BMSCs and the other groups. (**p*< 0.05)
